# Hereditary gynecological tumors associated with Peutz-Jeghers syndrome (Review)

**DOI:** 10.3892/ol.2013.1527

**Published:** 2013-08-16

**Authors:** KOUJI BANNO, IORI KISU, MEGUMI YANOKURA, KENTA MASUDA, ARISA UEKI, YUSUKE KOBAYASHI, AKIRA HIRASAWA, DAISUKE AOKI

**Affiliations:** Department of Obstetrics and Gynecology, School of Medicine, Keio University, Tokyo 160-8582, Japan

**Keywords:** Peutz-Jeghers syndrome, sex cord tumor, minimal deviation adenocarcinoma, lobular endocervical glandular hyperplasia, *STK11/LKB1*, endometrial cancer

## Abstract

Peutz-Jeghers syndrome (PJS) is an autosomal dominant disease that is characterized by gastrointestinal hamartomatous polyposis and mucocutaneous melanin spots. The tumor suppressor gene, *STK11/LKB1*, which is located on chromosome 19p13.3, has been reported to be responsible for this condition. PJS is complicated by benign and malignant tumors of various organs and complications from rare diseases, including sex cord tumor with annular tubules (SCTAT) and minimal deviation adenocarcinoma (MDA), which have also recently attracted attention in the field of gynecology. Among the total MDA cases, 10% are complications of PJS, and mutations in the *STK11* gene are closely associated with the development and prognosis of MDA. Furthermore, a new type of uterine cervical tumor, lobular endocervical glandular hyperplasia (LEGH), has been identified and has been predicted to be a precancerous lesion of MDA. The first case of LEGH induced by a germline *STK11* mutation has also been described. A high risk of endometrial cancer in PJS has also been reported. These developments suggest that PJS is an important syndrome of hereditary gynecological tumors that requires further study.

## 1. Introduction

Peutz-Jeghers syndrome (PJS) is an autosomal dominant disease that is characterized by hamartomatous polyposis in the gastrointestine and mucocutaneous melanin spots in the oral mucosa, lips, wings of the nose, fingers and toes. The first case was reported in a Dutch family by Peutz in 1921 (1). Jeghers *et al* described the details of another family in 1948 (2) and the syndrome was defined as an independent disease. The clinical features of PJS include gastrointestinal polyps that increase in number and size and the appearance of abdominal symptoms, including ileus and gastrointestinal hemorrhage. Among the types of hereditary gastrointestinal polyposis, the incidence of PJS is second highest following that of familial adenomatous polyposis. PJS develops at an incidence rate of 1 in 5–25×10^4^ individuals.

The tumor suppressor gene, *STK11/LKB1*, which is located on chromosome 19p13.3, is responsible for PJS. *STK11* is believed to be involved in cellular energy metabolism, cell proliferation, cell polarity, p53-dependent apoptosis, the regulation of vascular endothelial growth factor (VEGF) and Wnt signal transduction. Loss of heterozygosity (LOH) due to a mutation, including a deletion in the normal allele, in addition to a germline *STK11* mutation, may cause the clinical features of PJS, including gastrointestinal polyposis and cancerization of other organs. PJS is complicated by benign and malignant tumors of various organs and by rare diseases, including sex cord tumor with annular tubules (SCTAT) and minimal deviation adenocarcinoma (MDA). The development of endometrial carcinoma in patients with PJS has also attracted attention in the field of gynecology. Lobular endocervical glandular hyperplasia (LEGH) in a PJS patient with a germline *STK11* mutation has recently been reported, which has led to the hypothesis that LEGH is a prodromal lesion of MDA (3).

## 2. Clinical characteristics of PJS

A disease resembling PJS was described by Peutz in 1921 and by Jeghers *et al* in 1949 (1,2). The pathology of PJS is considered to be hamartomatous, since PJS polyps are not cancerous and their histology is similar to the normal mucosal structure. A more accurate description of the lesions is hyperplasia of the lacunar epithelium. However, the morphology differs from that of a general hyperplastic polyp. The gland opening is dilated toward the outer side due to the hyperplastic mucoepithelium and muscular fibers overgrow in a dendritic pattern along the epithelium. These changes are preceded by epithelial hyperplasia. Adjacent lamina muscularis mucosae are believed to bend and fuse to give the characteristic appearance and small lesions undergo hyperplastic changes (4,5).

Clinically, PJS is characterized by the development of abdominal symptoms, including pain, ileus and gastrointestinal hemorrhage, which occur with an increase in the number and size of the gastrointestinal polyps. The incidence of PJS is secondary to that of familial adenomatous polyposis among the types of hereditary gastrointestinal polyposis. Malignant and benign tumor complications may occur in PJS, with studies indicating that malignant tumors develop in approximately half of patients by the age of 57 years. The incidence of digestive organ malignant tumors is highest in the colorectum, followed by the stomach, small intestine, duodenum and pancreas. In regions other than the digestive organs, the incidence is reported to be highest in the uterine cervix, followed by the lung and ovary in female PJS patients (Table I) (3). Associations with ovarian SCTAT and uterine cervical LEGH and MDA have been highlighted in the field of gynecology, and an association with endometrial cancer has recently gained interest based on case reports and genetic studies (6–11).

## 3. *STK11/LKB1* and PJS

The gene that is responsible for causing PJS is the tumor suppressor gene, *STK11/LKB1*, which is located on the short arm of chromosome 19 (19p13.3) (12). The gene is 23 kb in size and is comprised of nine coding exons and one non-coding exon. The gene encodes a serine threonine kinase containing 433 amino acids (12). The mRNA is 3.0–3.3 kb in size and is expressed in almost all human tissues. Germline mutations of *STK11/LKB1* are observed in more than half of PJS patients. However, the somatic development of PJS in patients with no familial medical history and cases without a mutation in *STK11/LKB1* have also been described.

**Figure 1:**
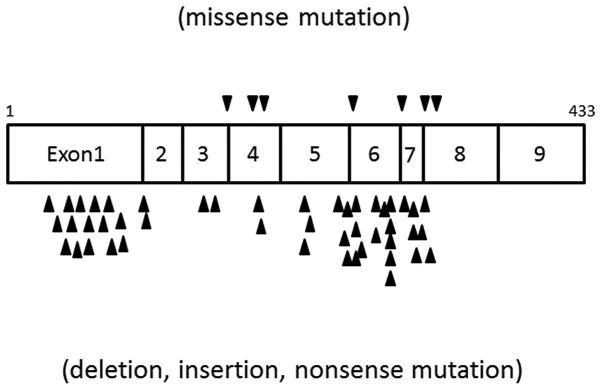
Germline mutations of the *STK11/LKB1* gene in patients with PJS (Peutz-Jeghers syndrome).

In 1997, Hemminki *et al* identified the responsible gene region in PJS to be near the chromosome 19 short arm marker using a linkage analysis of families with PJS (13). Amos *et al* confirmed this finding (14). The *STK11* gene was known to be present on chromosome 19 (15,16) and Yoon *et al* subsequently identified a number of mutation types in this gene, including missense, frame shift, nonsense and splicing site mutations, in 10 PJS patients using polymerase chain reaction-single strand conformation polymorphism (PCR-SSCP; Fig. 1) (17). Germline mutations were present in five of these patients. Tseng *et al* analyzed *STK11* mRNA expression in twin sisters with PJS with the same allele using PCR (18) and revealed that *STK11* gene expression was absent in the two subjects. These results suggest that *STK11* on chromosome 19 is responsible for PJS and that a mutation or decreased expression of *STK11* is the cause of the disease.

The protein product of *STK11* is involved in cellular energy metabolism, cell proliferation, cell polarity, p53-dependent apoptosis, the regulation of VEGF and Wnt signal transduction (3). LOH due to a mutation, including a deletion in the normal allele, in addition to the germline *STK11* mutation, results in gastrointestinal polyposis and cancerization of other organs, which are common clinical features of PSJ.

As noted previously, Yoon *et al* identified germline mutations in five of 10 PJS patients, which suggests other developmental mechanisms in the remaining five patients, based on *STK11* gene mutation-induced development (somatic case) and the association with other genes. Several studies have suggested the presence of a gene that is associated with PJS other than *STK11*. In an investigation of 21 PJS patients from 13 families, Papp *et al* identified that 8 (62%) of the 13 cases of PJS had familial medical histories and that the remaining five cases (38%) were due to *de novo* mutations (12). Germline mutations were screened in the 21 patients and 13 pathogenic mutations of *STK11* were identified. Three of these were frameshift mutations, three were nonsense mutations, two were mutations of the splicing sequence and five were deletions of exons 1–7. This deletion was noted in five of the 13 families, showing a high frequency, and was also shown to affect two genes that were located upstream of the *STK11* gene, *SBNO2* and *GPX4*, which are considered to modify *STK11*. This finding suggests that an abnormality in the genes that modify *STK11* function may promote the development of PJS, even in the absence of a mutation in *STK11* itself.

Souza *et al* initially reported a contiguous genetic syndrome in which a developmental disorder, heart malformation and facial dysplasia appear as phenotypes, in addition to the symptoms of PSJ (19). This syndrome is caused by a 19p13.3 chromosomal deletion of a region of ~1.1 Mb that includes *STK11*(19). Scollon *et al* also reported a syndrome with a similar gene deletion at a similar site, but the development of a cleft lip and gastrointestinal polyposis differed between the syndromes, suggesting that the phenotypes may vary, despite a common *STK11* deletion between the two syndromes (20).

## 4. SCTAT in PJS

Histologically, SCTAT is characterized by the annular growth of sex cord cells while hyaline bodies form around the nucleus. SCTAT includes ovarian Sertoli cell tumors, ovarian mucous/serous epithelial tumors and ovarian mature teratoma. Scully *et al* suggested that SCTAT is derived from ovarian granulosa cells and grows in a pattern that is characteristic of Sertoli cells (21). In another hypothesis, SCTAT has been considered to be comprised of sex cord-derived immature cells, which have the possibility to differentiate into granulosa and Sertoli cells. These tumors are positive for immunohistochemical staining of testosterone and estradiol and are diagnosed based on endocrinological symptoms in the majority of cases, including precocious puberty and irregular menstruation, which may involve amenorrhea, hypermenorrhea and postmenopausal hemorrhage (3).

SCTAT is an ovarian tumor that is most likely to complicate PJS, with ~36% of SCTAT cases reported to be associated with the syndrome (22). The clinical features of SCTAT differ between patients with and without PJS (22). In a comparison between SCTAT in 21 patients with PJS and 47 patients without PJS, Young *et al* identified that SCTAT with PJS was multifocal, bilateral, small and required microscopy to confirm the diagnosis in the majority of the cases. More than half of the cases were accompanied by calcification and the prognosis was favorable. In contrast, sporadic SCTAT was unilateral, large enough for palpation and calcified only in 12% of cases. In total, 20% of cases progressed to malignancy (22). Granulosa cell, Seltori-Lydig cell, borderline malignant and mucous tumors have been reported as other histological types of PJS-associated ovarian tumors, in addition to SCTAT.

## 5. MDA in PJS

MDA is a novel disease type that has been proposed for a condition that was previously termed adenoma malignum by Silverberg and Hurt in 1975 (23). MDA may be a subtype of adenocarcinoma, but it has a well-differentiated histology that is indistinguishable from that of the normal cervical gland. Certain patients that are diagnosed with MDA have a favorable prognosis, and in 1999, Nucci *et al* proposed the disease type lobular endocervical glandular hyperplasia (LEGH) for these cases (24). LEGH has been assumed to be a precancerous lesion of MDA in one study; however, a conclusion has not been established until recently (6).

MDA is a well-known gynecological tumor that is likely to complicate PJS. The incidence of MDA in PJS patients is estimated to be 15–30%, while ~10% of MDA cases are complicated by PJS. The mean age of MDA patients with PJS has been reported to be 33 years old, whereas that of MDA patients without PJS is 55 years old, indicating a young onset age for the dual disease (25). MDA complicating PJS is considered to be a malignant tumor with the poorest prognosis among all the gynecological tumors complicating PJS.

A mutation in the gene that is responsible for PJS, *STK11*, and LOH on chromosome 19p13.3, the location of *STK11*, have been analyzed in studies of MDA without PJS (26,27). LOH at 19p13.3 was identified in ~50% of these cases. In another study, an allelic deletion of >3.5 Mb was noted on chromosome 19p13.3 in nine MDA patients without PJS characteristics (28). Furthermore, in an investigation of the association between *STK11* mutation and LOH on chromosome 19p13.3, Connolly *et al* identified no somatic mutation in the *STK11* coding region in eight MDA patients without PJS, but LOH was present on chromosome 19p13.3 in three of those patients. Therefore, somatic mutations in *STK11* are absent in MDA without PJS, but LOH may occur at chromosome 19p13.3 (25). These results demonstrate that LOH may occur at chromosome 19p13.3, the location of the *STK11* gene that is responsible for PJS, in certain MDA patients without PJS, suggesting the involvement of an unknown tumor suppressor gene on chromosome 19p13.3. Lee *et al* also identified LOH on the chromosome on which *STK11* is present in six of nine MDA patients without PJS and at a site 190 kb away from *STK11* in two patients. These findings also suggest the presence of a tumor suppressor gene that is associated with the development of MDA, other than *STK11*, on chromosome 19p13.3. Linkage with 19q13.4 has also been reported (29).

Kuragaki *et al*(27) identified *STK11* mutations in six out of 11 MDA patients without PJS and also confirmed LOH in all six of the patients with *STK11* mutations, suggesting that *STK11* on 19p13.3 is associated with MDA, in addition to PJS. With regard to the presence of the *STK11* mutations in only six of the 11 patients with mucous MDA, Kuragaki *et al* suggested that the development of mucous MDA involves the loss of MDA expression, the post-translational modification of the STK11 protein and a gene other than *STK11*(27). Further analysis of *STK11* at the exon level in the six MDA patients with *STK11* mutations revealed that a mutation was present in the exons of one out of three of the patients (27). In other studies, Nakagawa *et al* identified a germline mutation in exon six of *STK11* in five out of ten PJS patients (30), Connolly *et al* observed mutations in exons 4 and 6 in two SCTAT patients with PJS (25) and Hemminki *et al* identified a mutation in exon 1 in seven out of 12 PJS patients (29). These results suggest that the significant exons of *STK11* for PJS are exons 1, 4 and 6.

Hirasawa *et al* recently described the first case of LEGH in a PJS patient with a germline *STK11* mutation. The mutation was a deletion of four bases at codon 263 of exon 6 (6). This is significant in terms of the development of LEGH as a uterine cervical tumor in a PJS patient and also with regard to LEGH as a potential precancerous lesion of MDA.

The prognosis of MDA has been reported to vary between being extremely poor (31) to being mostly equal to that of well-differentiated uterine cervical adenocarcinoma of the same stage (32). Kuragaki *et al* investigated the association between *STK11* mutations and prognosis in 11 MDA patients without PJS (27). The clinical stages of the patients were IB and IIB in five and one out of six patients with *STK11* mutations, respectively, and IB and IIB in four and one out of five patients with no *STK11* mutations, respectively, showing no difference in the clinical stage between the two groups. However, four of the six patients with *STK11* mutations succumbed within 24 months following surgery and the tumor recurred in one of the two surviving patients. In contrast, all five of the patients without *STK11* mutations survived for >50 months following surgery, suggesting that the prognosis of MDA with *STK11* mutations is poorer than that of MDA without *STK11* mutations (P=0.039). The prognosis of PJS patients whose condition is complicated by MDA has also been recorded to be poor in numerous other studies (3,33). This may have been due to a higher level of *STK11* mutations in the MDA patients with PJS than in those without PJS.

## 6. PJS and endometrial carcinoma

PJS is characterized by the development of numerous gastrointestinal hamartomatous polyps and mucocutaneous pigmentation and a higher risk of malignant tumors in the digestive tract and other organs compared with the general population. The risk of gynecological cancer is also high, with the lifetime risk of endometrial carcinoma in females with PJS reported to be 9% (34). However, this risk in PJS patients is lower than that of patients with Lynch syndrome and surveillance for endometrial carcinoma in PJS patients is considered unnecessary, unlike for ovarian, cervical and breast cancers (35).

## 7. Conclusion

Benign or malignant tumors readily develop in organs such as the digestive tract, mammary glands, ovaries and uterus in PJS patients, with a risk that is 10–18 times higher than that of the general population (36). In females with PJS, the incidence of two rare gynecological tumors, SCTAT and MDA, also tends to increase. In a few patients, several gynecological tumors may complicate PJS simultaneously. One example of this may be observed in the female PJS patient described by Mangili *et al*, in whom microscopic ovarian SCTAT and uterine cervical MDA were identified (37).

With regard to the association between MDA and PJS, *STK11* on chromosome 19p13.3 is likely to be the gene responsible for the two diseases. Gene analyses have been performed separately for PJS and MDA, but only case reports are available for MDA patients with PJS and no comparative gene analysis has been described. Until recently, there have also been no studies of concomitant PJS and LEGH, which is considered to be a precancerous lesion of MDA (6).

The clarification of the association between PJS and LEGH, MDA, SCTAT and endometrial carcinoma requires the elucidation of the developmental mechanism at the genetic level. However, PJS is a rare disease and patients with SCTAT and MDA complications are rarer, making the number of patients very small. A complete gene analysis requires a large number of patients and will require a multicenter study. However, probing of the genetic mechanisms of SCTAT and MDA complicated by PJS may lead to greater understanding of the developmental mechanisms of uterine cervical adenocarcinoma and simultaneous multiple gynecological tumors that complicate PJS. Further studies are anticipated in this field.

## Figures and Tables

**Table I:** Cancer cases reported in patients with PJS.

Location	Number of patients
Gastrointestinal	
Esophagus	1
Stomach	16
Small intestine	22
Large intestine	26
Pancreas	8
Extraintestinal	
Breast	17
Uterine cervix	10
Ovary	7
Uterus	2
Fallopian tube	1
Testis	1
Prostate	1
Lung	9
Thyroid	2
Leiomyosarcoma	2
Gall bladder	1
Liver	1
Basal cell	1
Osteosarcoma	1
Multiple myeloma	1

PJS, Peutz-Jeghers syndrome.
